# Implementation of an experimental isolated lung perfusion model on surgically resected human lobes

**DOI:** 10.1038/s41598-019-48719-8

**Published:** 2019-08-21

**Authors:** Alexis Slama, Christian Raber, Celia Hedderich, Paul Stockhammer, Balazs Hegedüs, Achim Koch, Dirk Theegarten, Till Ploenes, Clemens Aigner

**Affiliations:** 1 Department of Thoracic Surgery and Thoracic Endoscopy, University Medicine Essen-Ruhrlandklinik, Essen, Germany; 2 Department of Pathology, University Medicine Essen-Ruhrlandklinik, Essen, Germany; 3 Department of Thoracic and Cardiovascular Surgery, West German Heart and Vascular Center Essen, University Hospital Essen, Essen, Germany; 4 Division of Thoracic Surgery, Department of Surgery, Comprehensive Cancer Center, Medical University of Vienna, Waehringer Guertel 18–20, A-1090 Vienna, Austria

**Keywords:** Experimental models of disease, Translational research

## Abstract

Isolated lung perfusion (ILP) is an ideal model to study treatment effects on a variety of pathologies. As published research mostly relies on rejected donor lungs or animal organs, this study investigates the use of surgically resected human lobes as an alternative and novel model for personalized experimental research. Ten surgically resected lobes were perfused in acellular and normothermic condition. The indication for surgery was lung cancer. Perfusion and ventilation were adapted to the size of the lobes and both functional and metabolic parameters were assessed during ILP. Patients (age 67.5 y (59–81)|♀n = 3|♂n = 7) underwent anatomic pulmonary lobectomy. Ischemic time between arterial ligation and ILP was 226 minutes (161–525). Median duration of ILP was 135 (87–366) minutes. Gas exchange and mechanical respiratory parameters remained steady during ILP (pulmonary venous pO_2_ 196(151–219) mmHg | peak AWP: 14.5(11–22) cmH_2_O). Metabolism stayed constant during ILP (Glucose consumption: 1.86 mg/min/L_TLC_ (95%CI: −2.09 to −1.63) | lactate production: 0.005 mmol/min/ L_TLC_ (95%CI: 0.004 to 0.007)). ILP of surgically resected human lobes is a feasible and promising method. By maintaining a near physiological setting, this model may pave the way for future experimental lung research including cancer research, transplantation, physiology, pharmacology and mechanical ventilation.

## Introduction

Lung physiology and therapeutic interventions can ideally be studied on isolated lung models. Historically the first experiments on (out of body) isolated and perfused lungs (isolated lung perfusion = ILP) were performed and published only in the seventies^1–3^. Despite rudimentary perfusion strategies resulting in tissue damage and therefore short perfusion times, researchers early recognized the potential use of ILP within the field of organ preservation and transplantation^4^. The major clinical breakthrough was made after intense basic and translational research in 2001 with the first successfully transplanted human lung after *ex-vivo* lung perfusion (=EVLP)^5^. This has been expanded and EVLP is now widely used to re-assess marginal donor lungs and has been studied as preservation technique for standard donor lungs. However, all clinical applications of isolated organ perfusion are based on the perfusion of either a double lung block or entire single lungs. Less is known about perfusion of isolated lobes and its applicability for research, particularly in an oncologic setting with tumor affected lobes. Thus, we aimed to establish an *ex vivo* perfusion model of isolated resected human lobes based on perfusion protocols in use for entire lungs which can be used as a research platform for several applications including *in-vivo* lung perfusion.

## Development of ***ex-vivo*** Lung Perfusion in Transplantation

The first “modern” *ex-vivo* lung perfusion (EVLP) protocol was initially developed to assess gas exchange in donor lungs retrieved from organ donors without cardiocirculatory function (donation after circulatory death = DCD)^5–7^. This method used a novel hyper-oncotic albumin-based perfusion solution (Steen Solution®), which allowed for the first time a prolonged functional re-evaluation of lungs with unclear or initially insufficient function (defined as “marginal” donor lungs). After the first successful clinical lung transplantation (LuTX) of such a “re-conditioned” marginal donor lung in 2007^8–10^, this new approach led to a tremendous worldwide interest. Considering the increasing organ demand and their limited availability, EVLP has been the most recent development with the potential to reducing waiting list mortality. Soon, a new (acellular) perfusion approach was developed in Toronto^11,12^, which led to the first published clinical series of 23 EVLPs of initially rejected donor lungs which were successfully transplanted after re-evaluation^13^. Despite different perfusion approaches (acellular and blood-based), all following reports (2012 Vienna^14,15^, 2012 Gothenburg^16,17^, 2012 Harefield^18^, 2012 Newcastle^19^, 2014 Milan^20^, 2014 Paris^21^) described comparable post-operative outcomes between LuTX patients receiving an EVLP lung (initially classified as unsuitable) and those who received a “standard” donor lung. Thus, the safety and feasibility of EVLP to increase the donor lung pool available for transplantation by re-evaluating “marginal” donor lungs could be clearly demonstrated. Those promising results led to two randomized trials on “standard” lungs^22,23^. These showed that the short timeframe between retrieval of a donor lung and its reperfusion within the recipient could be significantly prolonged (>4 hours) without altering organ quality during EVLP. In addition, postoperative results including oxygenation, primary graft dysfunction, intubation time, duration of hospitalization were comparable between perfused lung transplantations and controls. Of note, another advantage of this method was to identify initially unrecognized impairments of the grafts before transplanting them, and thereby to prevent potential risk of the recipients. Different mechanisms and strategies are essential for prolonged EVLP. On the one hand the hyper-oncotic albumin/dextran perfusate reduces the formation of tissue edema leading to reported preservation times of over 12 hours^24^. On the other hand, a near physiologic setting with regard to perfusion and ventilation allows for recruitment of atelectatic lung tissue and thereby to a reduction of a ventilation perfusion mismatch within the graft^8^. Also, thrombi and pro-inflammatory cytokines can potentially be flushed out during perfusion^25^.

## ***In-vivo*** Lung Perfusion

Although *in vivo* lung perfusion (IVLP) with chemotherapeutic drugs still has to prove its clinical benefit in thoracic oncology, promising future developments of this method can be expected. During such procedure, pulmonary circulation is separated unilaterally and reattached to an extracorporeal circuit without removing the lungs from the patient. In this context, high-dose chemotherapy or immunotherapy can be locally administered to malignant lung diseases, especially lung metastases, without provoking systemic drug-related side effects of the patient. In the first clinical series^26^, patients with unresectable tumors (n = 4 sarcoma metastases and n = 4 pulmonary metastasized bronchoalveolar cancer) underwent IVLP with doxorubicin or cisplatin. During a perfusion time of over 45 minutes a high concentration of the substance was measured within lung tissue whereas the concentration in the systemic circulation remained below the toxicity threshold. None of the patients showed treatment related adverse events however no tumor response was observed. A porcine experiment (n = 6) showed that IVLP with Steen solution can be performed for total duration of four hours without histological or functional damage to the lung^27^. Those observations could be reproduced with the addition of doxorubicin and Ifosfamide^28^. So far, dose finding for therapeutic IVLP has been carried in toxicity/efficacy experiments on perfused (porcine and human) lungs and *in vitro*^28–30^.

## Experimental ILP on Surgically Resected Lobes

To our best knowledge, only one single model of isolated lung perfusion on surgically resected lobes (for bronchial carcinoma) has been published so far^4^. In that study, lung lobes were perfused for up to 180 minutes with whole blood and saline, which ultimately led to pronounced lung edema. Importantly, subsequent histologically examination of the tissue showed no interference of ILP on pathohistological grading and staging^31^. The model was first used to study pharmacokinetics and toxicity of anti-cancer drugs within the lungs^31,32^. Although the feasibility of normothermic lung perfusion has been clearly demonstrated on porcine, murine and human lungs^33^, there is still a lack of highly reproducible and cost- but also resource-effective models. Thus, this study suggests a novel method of normothermic isolated human lobar lung perfusion for future experimental research.

## Methods

Approval was obtained from the ethics committee of the university of Duisburg-Essen before first patient enrollment (Nr 17-7802-BO). The study was registered on the German register for clinical trials (DRKS00013927).

Informed consent form was obtained from all included patients. In detail, patients who were scheduled for lobectomy and who met the inclusion criteria (Table 1) were informed prior surgery about the possibility to donate their resected lobe to be included in this study. All experiments were performed according to the principles of the Declaration of Helsinki. All relevant guidelines and regulations were met.

**Table 1:** Patient and organ specific inclusion criteria for isolated lung perfusion.

Anatomical resection of one or multiple lobes
Given informed consent
Absence of infectious disease (HIV, HBV, HCV, TBC, MRSA, MRGN)
Absence of recent irradiation or chemotherapeutic treatment (14 days)
Absence of purulent infiltrates
Absence of bullous emphysema
Absence of parenchymal and pleural damage

## Preparation of the Lobe

**Figure 1:**
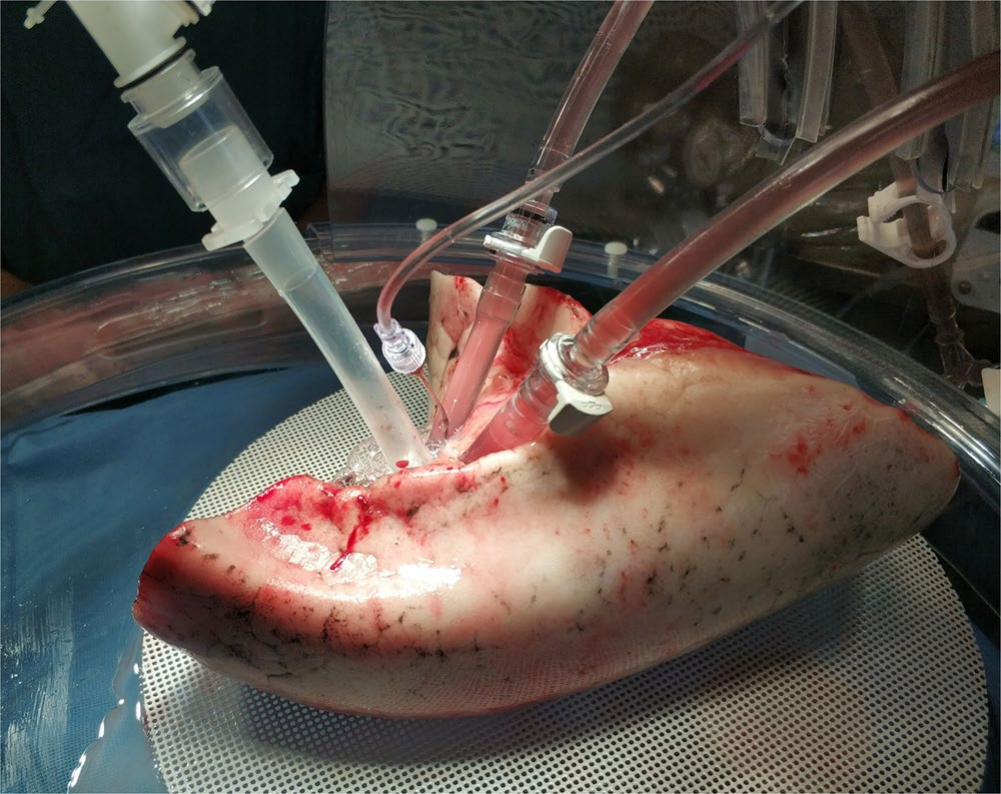
Left lower lobe with three cannulas and a pressure probe in place during ILP.

Surgery was performed according to our clinical routine either via open surgery (anterolateral thoracotomy) or a minimally invasive approach (VATS: video assisted thoracoscopic surgery or RATS, robotic assisted thoracic surgery). After retrieval of the pulmonary lobe it was immediately submerged in cold saline. All ligated or stapled vessels were reopened to evacuate blood and possible thrombi. Subsequently the vasculature was flushed both antegrade (via the segmental arteries) and retrograde (via the pulmonary vein) with 1 liter of pH-buffered Perfadex® solution. At this point the lobes where meticulously inspected for any iatrogenic damages or insufficient cold perfusion. Lobes that were unsuitable for perfusion were directly sent to pathology. For included lobes, bronchi were reopened if they had been closed with a stapler device during surgery and resection margins were cut down if a frozen section was necessary to evaluate surgical-oncological radicality of the resection. Then, all vascular and bronchial stumps were anastomosed on silicone tubing of the same diameter (referred to as cannulas) with running sutures (Prolene 4/0 & 5/0). Separated arteria and veins were reconnected either by anastomoses or by the use of Y-connectors (Fig. 1). The bronchial cannula was clamped and the lobes were stored in the fridge (4 °C) in a closed bag filled with Perfadex.

## ILP Technique

ILP was carried out similar to our method used for EVLP in clinical routine^34^. The general technique for clinical acellular EVLP has been slightly adapted by different centers after its first publication and we will herewith focus on the differences and the specificities of our experimental setting.

A custom assembled perfusion circuit (1/4^th^ inch inner diameter) has been used in this series. The lobes were placed in a commercially available Xvivo™ chamber. The pulmonary vein drained into the main reservoir (Maquet cardiotomy reservoir). From here a centrifugal ECMO pump (Maquet cardiohelp) pumps the perfusate through a build in heat exchanger and a membrane oxygenator (used for de-oxygenation and CO_2_ addition) before recirculating it through the lungs via the arterial cannulas. Ventilation was achieved with pediatric sized tubings and OR-Ventilators (Dräger Primus, Dräger Zeus). An additional roller pump (Kamoer Lab UIP) was used to recirculate any perfusate leaking in the chamber. The pressure from the arterial cannula was measured with a button cannula placed within the pulmonary artery and a standard OR pressure transducer (smiths medical LogiCal®).

The circuit was primed with 1 L of Steen™ Solution to which cortisone, heparin and antibiotics were added. pH was buffered (>7.0) by subsequent addition of Bicarbonate or Tris (THAM) to the perfusate.

Perfusion and ventilation parameters were calculated according to the size and the weight of the patient as well as the resected lobe. After a short retrograde de-airing of the vasculature, the lobes were slowly warmed up and perfusion flow was gradually increased during the first hour up to a calculated maximum flow (estimated on patient cardiac output and size of the resected lobe; Table 2). When normothermia (35–37 °C) was reached after the first 20 minutes, ventilation was started and the perfusate was de-oxygenated. In contrast to clinical protocols where an hourly evaluation is performed (at FiO_2_:1.0, VT:10 ml/kg, frequency: 10 bpm), ventilation settings were not changed during perfusion and FiO_2_ was set to 0.4. Perfusion and ventilation settings are summarized in Table 2.

**Table 2:** Formulas and settings for ILP; CO: predicted cardiac output; IBW: ideal body weight; BPM: breaths per minute; PEEP: positive end expiratory pressure.

Flow	Flow(max) = calculated CO × 0,4 × (number of segments/19)
Ventilation	VT = 7 × IBW × (number of segments/19)
	FiO_2_: 0,4
	Frequency: 8 BPM
	I/E ratio = ½
	PEEP 7–8 cmH_2_O

## Data Handling

During ILP, physiologic data, perfusion gas exchange and glucose metabolism were assessed and recorded every 15 minutes. Blood gas analysis was performed with a commercially available blood gas analyzer (Radiometer ABL90 and ABL800). For later analysis, tissue samples were taken and preserved on formaldehyde, before and at the end of ILP. Perfusate samples were collected every 15 minutes during ILP.

After ILP, specimens were sent to pathology for clinical routine examination and TNM staging (UICC 8^th^ Edition 2016).

All data was documented by using Microsoft Excel. Analyses and graph plottings were performed in Graphpad Prism Graphad Prism (Graphpad Software, Version 8.0, San Diego, CA, USA). If not stated otherwise data is presented as median and range.

## Results

From March 2018 to September 2018, 26 patients with suspected (n = 13; 50%) or verified (n = 13; 50%) malignancy of the lungs were screened for eligibility and gave informed consent to this study. Of those, 17 (65.4%) ultimately underwent surgical lobectomy. Finally, 10 of these resected lobes were included in this trial after cold perfusion and visual inspection. The other 7 lobes were not included either due to anatomical (unsuitable for cannulation due to short stumps or centrally located tumors requiring frozen section analysis) or iatrogenic (lesions of the pleura or parenchyma during the retrieval of the specimen).

Table 3 summarizes all patient and perfusion related data. Patients had a median age of 67.5 (59–81) years and 3 were female (30%). 7 patients had a history of smoking (35 pack years (15–75)) and 5 of those had a diagnosed lung emphysema. Median BMI was 26.4 (18.8–33.7) and estimated cardiac output was 5.9 (4.1–6.5) L/min. Predicted total lung capacity (TLCp) was 7.4 (5.0–8.2) L whereas plethysmography lung volumes (TLCr) were higher (8.1 (5.4–9.3) L).

**Table 3:** Patient and perfusion summary; LLL: left lower lobe; RUL: right upper lobe; RLL: right lower lobe; RML: right middle lobe; NET: neuro-endocrine tumor; ACA: adenocarcinoma; SCC: squamous-cell carcinoma; TLC: typical lung carcinoid.

case nr.	1	2	3	4	5	6	7	8	9	10	median (range)
age	68	69	59	80	67	65	66	65	78	81	67.5 (59–81)
gender	m	f	m	f	m	m	m	m	m	f	
emphysema	yes	yes	no	no	no	no	yes	yes	yes	no	
surgery type	RATS	thoracotomy	thoracotomy	VATS	VATS uniportal	thoracotomy	thoracotomy	VATS	thoracotomy	thoracotomy	
lobe	LLL	RUL	LLL	LLL	RLL	RLL	LLL	LLL	LLL	RML	
wedge resection			yes	yes							
histopathology	NET	ACA	SCC	ACA	TLC	ACA	ACA	SCC	pneumonia	TLC	
TNM staging and grading	pT3 pN0 L0 G3 R0	pT1c pN2 L1 R0	pT1b pN0 G3 L0 V0 R0	pT2a pN0 G3	pT1b pN0 G1 L0 V0 R0	pT2a pN1b G3 L1 V1 R0	pT2b pN2b1 G3 L0 V0 R0	pT2 pN0 G2 L0 V0 R0		pT3 pN0 G1 L0 V0 R0	
perfused segments	4	4	3.5	3.5	5	5	4	4	4	2	4 (2–5)
arterial cannulas	2	2	1	3	2	1	2	2	2	1	2 (1–3)
venous cannulas	1	1	1	1	1	1	1	1	1	1	1 (1)
warm ischemic time (WIT)	45	62	60	33	60	46	123	35	95	12	53 (12–123)
cold ischemic time (CIT)	204	147	175	175	383	220	47	490	66	206	189 (47–490)
total ischemic time (TIT)	249	209	235	208	443	266	170	525	161	218	226 (161–525)
duration of ILP	92	163	152	90	118	210	209	88	87	366	135 (87–366)

None of the patients had neo-adjuvant chemotherapy or radiotherapy. 6 patients had open surgery (thoracotomy) whereas the other 4 patients were operated by minimally invasive techniques (3xVATS, 1xRATS). 2 patients had an extra-anatomical wedge resection to confirm malignancy by frozen section of the specimen, followed by lobectomy. 4 of the perfused lobes were from the right lung (1x upper lobe, 1x middle lobe, 2x lower lobe) and the remaining six were left lower lobes.

Median time between arterial clamping of the lobe within the patient and cold storage was 53 (12–123) minutes (WIT: warm ischemic time). After cool down, the median time until the start of ILP was 189 (47–490) minutes (CIT: cold ischemic time). Total ischemic time (TIT) before ILP was 226 (161–525) minutes. Cannulation was performed after the cold preservation with up to three arterial limbs and a single venous anastomosis in all cases. ILP was continued until no perfusate was left in the venous reservoir (due to extravascular loss and evaporation over the ventilator and the oxygenator). Median duration of ILP was 135 (87–366) minutes.

Histopathological evaluation after ILP revealed that 9 out of 10 resected lobes harbored primary lung cancer. Only one case was carnifying pneumonia. Importantly, tumor staging remained unaffected by the preceding ILP. Malignancies were adenocarcinoma (n = 4), squamous cell carcinoma (n = 2), typical lung carcinoid (n = 2) and a neuroendocrine tumor of the lung (n = 1). Patient and perfusion data are summarized in Table 3.

**Figure 2:**
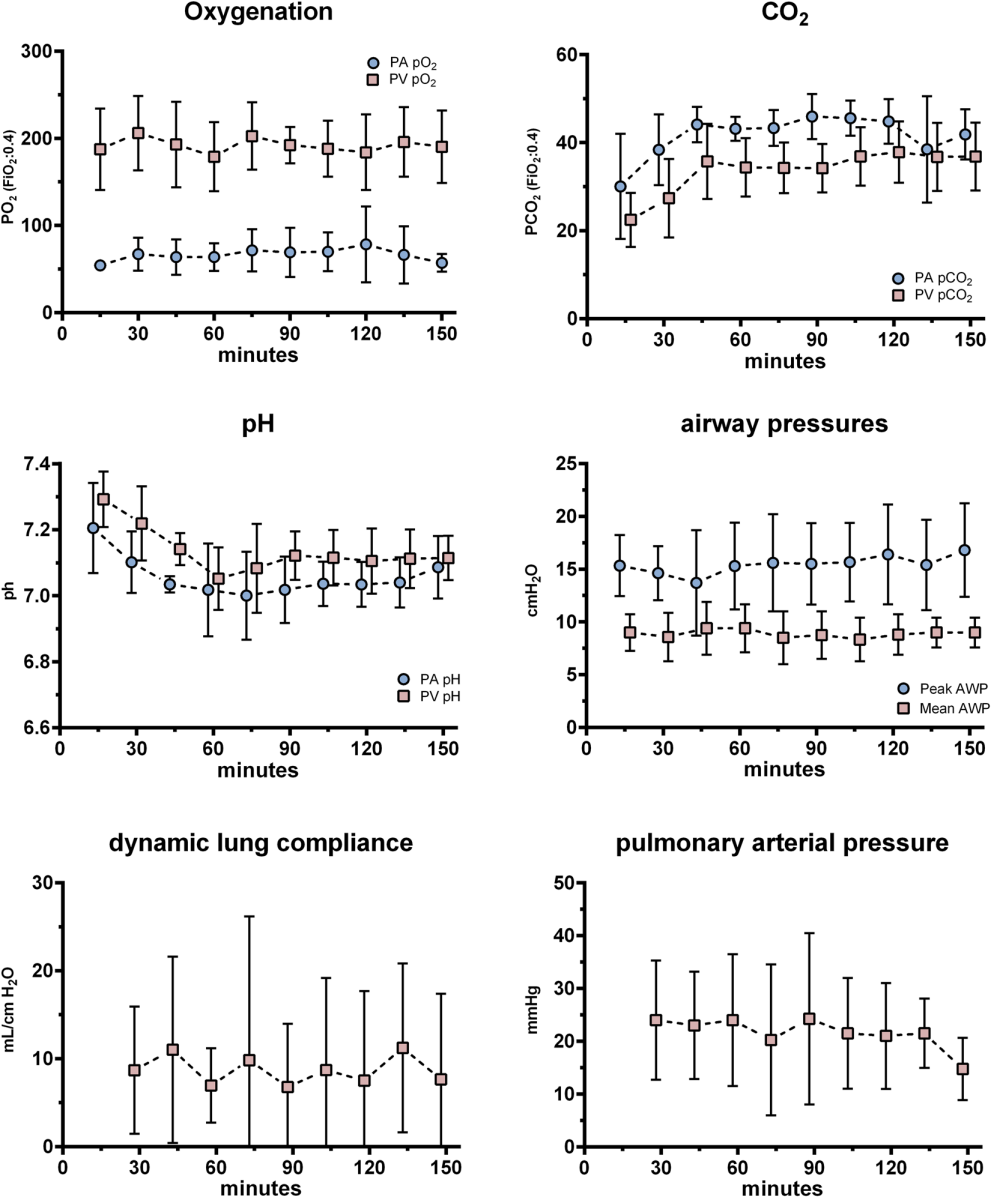
Functional parameter during ILP (box and whiskers expressed as mean/SD; PA: pulmonary artery; PV: pulmonary vein; AWP: airway pressure.

During ILP and after initiating ventilation, O2 and Co2 partial pressures remained steady over time in both arterial and venous measurements. The observed increase of CO2 in the first 30 minutes of ILP can be explained by the lack of sweep gas over the ECMO membrane during the warm-up phase. Ventilation parameters such as airway pressures and dynamic lung compliance showed no significant changes over time. pH decreased steadily and was buffered with the addition of bicarbonate to a value above 7.0 (Table 4; Fig. 2).

**Table 4:** Functional parameters during ILP; PV: pulmonary venous; PA: pulmonary arterial; AWP: airway pressure; p values are computed in F-tests of the linear regression model.

	60 min	90 min	120 min	changes over time (linear regression; *p*)
PV pO_2_ (mmHg)	188 (97.3–224)	196 (151–219)	201.5 (129–229)	**0.696**
PA pO_2_ (mmHg)	61.15 (47–100)	59.75 (46.2–130)	58.9 (52.4–155)	**0.773**
PV pCO_2_ (mmHg)	33.2 (27.8–45)	34.2 (26.6–43)	38.7 (28–46)	**0.0005**
PA pCO_2_ (mmHg)	43.1 (38.3–47)	44.2 (39.4–53.6)	46 (38.6–50.6)	**0.027**
PV pH	7.065 (6.89–7.17)	7.13 (6.99–7.21)	7.085 (7.0–7.24)	**0.004**
PA pH	7.025 (6.78–7.24)	7.03 (6.85–7.15)	7.0 (6.99–7.15)	**0.073**
Mean AWP (cmH_2_O)	9.5 (5–12)	8.5 (5–12)	8 (7–12)	**0.746**
Peak AWP (cmH_2_O)	15.5 (8–21)	14.5 (11–22)	16 (12–24)	**0.194**
lactic acid (mmol)	0.478 (0.2–1.17)	0.584 (0.268–1.094)	0.73 (0.327–1.294)	**<0.0001**
glucose consumption (mg)	303.6 (593–122)	362 (414–45)	398 (530–27)	**<0.0001**

**Figure 3:**
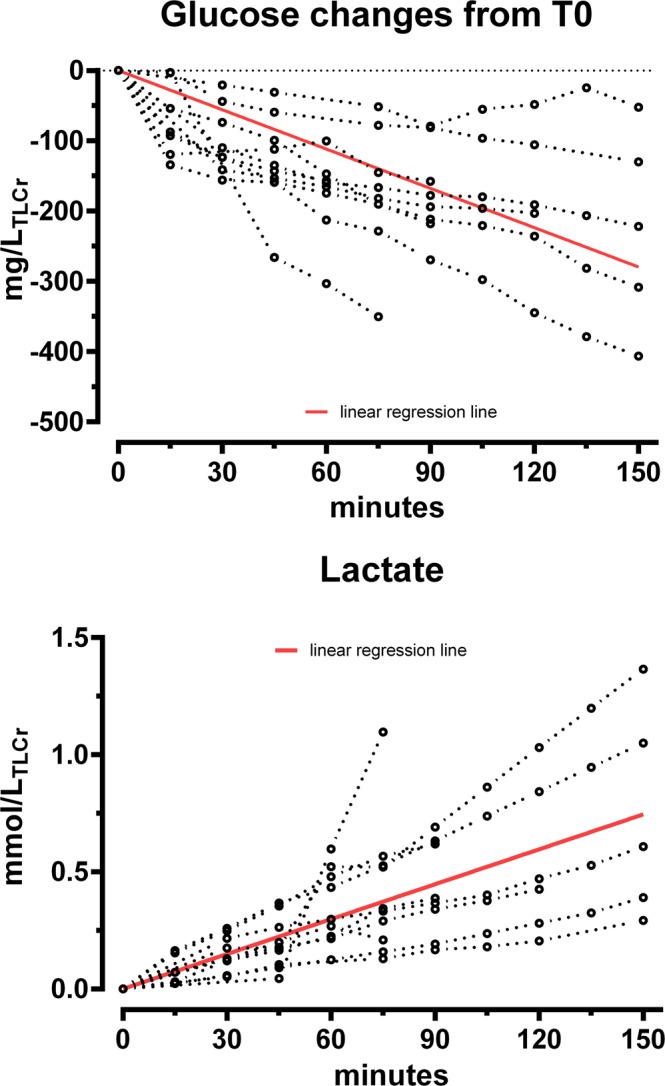
Glucose consumption and lactate production. Each replicate is presented. Red line: linear regression estimate; TLCr: measured total lung capacity.

Measurements of lactate and glucose within the perfusate demonstrated the metabolic activity of the perfused tissue. The changes over time were mostly linear. In order to adapt for different lobe sizes and hence to compare the metabolic state of the different cases, data was indexed on the TLC of the patients. In a linear regression model, the best fit line estimated a glucose consumption of 1.86 mg/min/L_TLC_ (95% CI: −2.09 to −1.63) and a lactate production of 0.005 mmol/min/ L_TLC_ (95% CI: 0.004 to 0.007) (Fig. 3).

## Discussion

The approach of a normothermic ventilated and perfused lung or lung lobe (either animal or cadaveric) has been initially only used with various degrees of success in physiological and pharmacological experiments^4,35,36^. Prior to the recent major breakthroughs of EVLP in clinical lung transplantation, the technique had been refined to enable a preserved physiologic graft function until reimplantation within the recipient^7,8,12^. The goal of this study was to translate that clinical knowledge on lung perfusion to a novel and reproducible experimental human model which may be thereafter used for future preclinical approaches.

The advantages of ILP on resected human lobes over its alternatives are numerous. First, research on cadaveric lungs, either from deceased hospitalized patients or from organ donors (with lungs unsuitable for LuTX) is highly restricted^33^. The limitations are given by high costs (non-local organ explanation), difficult logistics (short notice and organ retrievals taking place mostly during the night), legal provisions (need for consent, autopsy)^37^ and time dependent ischemic damage of the tissue^38,39^. In contrary, all these restraints do not apply for surgically resected lobes as patients scheduled for surgery are asked for consent, their procurement bears no additional cost or risk and the warm ischemic time can be reduced to a minimum, thus preserving tissue vitality. Secondly, there is widespread ethical agreement that the number of animal experiments should be limited to prevent unnecessary animal harm and suffering^40^. Apart from that, animal experiments are highly expensive. Of note, it can be safely assumed that an isolated human lobe will give more comparability to human preclinical and clinical questions than experiments performed in lung tissue from a different species.

Another major advantage is that surgically resected human lobes contain specific and known pathologies. Thereby, malignancies or structural diseases of the lung (including lung cancer, metastasis, emphysema, fibrosis, bronchiectasis, pulmonary hypertension) as well as surrounding healthy parenchyma can be investigated in a near physiological setting. The isolation of a lung lobe from any other organ system and the possibility to interfere with ventilation, circulation and the composition of the solution allows for reproduceable and precise measurements of functional parameters. Also, biomolecular processes and cell migration (e.g. free-floating cancer cells) could potentially be assessed in detail in future approaches^41–43^.

This pilot study gives the results and observation of our first 10 ILP experiments with resected human lobes. Since the ILP protocol was built upon clinical expertise, the first encountered pitfalls were specific to the surgical technique and not to the perfusion process itself.

First, the time between clamping of the arteries and the actual removal of the specimen can vary tremendously. This can be attributed to variations in anatomy, to different surgical approaches (VATS or thoracotomy) and to the individual surgeon experience. The preservation of the lobe by flushing and cooling can only take place after having retrieval from the patient. Thus, warm ischemic damage can only be reduced by shortening this period. Secondly, in minimally invasive surgical approaches (VATS and RATS), the deflated lobe has to be extracted from the chest cavity through a very small incision diligently to keep the fragile lung tissue intact during retrieval.

Thirdly, the number of vascular and bronchial stumps as well as their length varies on the technique used to seal and divide them. When using a mechanical stapling device, routinely applied in minimally invasive surgery, the portion of the vessel or bronchi secured with staples (~3 mm) has to be shortened to suture a cannula on. If that suture line happens to be at a vascular intersection or a bronchial carina, those vessels or bronchi have to be anastomosed together, which is technically challenging and can subsequently lead to a perfusion mismatch on a segmental level.

Although theoretically all pulmonary lobes can be cannulated for ILP, it is substantially more difficult and time consuming for upper lobes. The multitude of variation in the branching pattern of the main pulmonary artery into their segmental arteries necessitates different approaches to rebuild a common arterial trunk. However, in lower lobes with sufficient stump length, only the common basal artery and proximal to it the superior segmental artery (segment 6) have to be connected together and sutured to the cannula.

All these challenges in the preparation process for ILP restricted us to use only 10 out of 17 lobes. The other 7 lobes had to be excluded due to insufficient vascular stumps or obvious tears in the parenchyma.

In terms of learning curve, our cannulation technique improved gradually which ultimately led to shorter ischemic periods. In our series, we could even observe that the two lobes with the longest stable perfusion needed only one arterial cannula and had comparable short warm ischemic periods.

Presumably, the cumulative ischemia/reperfusion damage undergone by a resected lobe between arterial clamping and ILP will strongly affect the tissue integrity and thereby the possible duration of ILP. Following circumstances have to be mentioned: the first lobe in this series was resected by means of robotic assisted lobectomy (RATS). Only after the beginning of ILP, this lobe was shown to have been traumatized during preparation and during retrieval from the chest cavity which ultimately led to pronounced edema.

Because of logistical reasons, another lobe (#8) was subjected to a total ischemic time of 525 minutes during which it completely deflated because of an unrecognized pleural lesion. Consecutively the performance was rather poor with a reduced perfusion time of 88 minutes.

In a third case (#9), definitive histology revealed a carnifying pneumonia. Right from the start of ILP, an intraparenchymal broncho-vascular fistula was recognized. Ultimately, the fluid overflow into the airways made ventilation impossible within the first hour of perfusion.

From a physiological point of view, our experiments were limited by edema formation during ILP. Although all functional parameters remained stable during perfusion, the lungs became progressively edematous and the solution within the circuit decreased steadily until the reservoir run on empty. Neglecting fluid loss via evaporation (breathing, ECMO membrane and uncovered pleural surface) this fluid leakage into the parenchyma without imminent functional impairment in the first hours of perfusion has been observed previously in clinical EVLP of whole lungs^44^ and in experimental ILP with both acellular and cellular perfusion solution^4^. Presumably, the edema formation is caused by an altered capillary integrity (as a result of ischemia/reperfusion damage) leading to extravascular shifting of colloids (albumin and dextran) and thereby fluid. In lobes affected by malignancy or pneumonia it is conceivable that chronic inflammation also contributes to a capillary leak and possibly to the release of inflammatory cytokines. By putting our focus on the technical challenges and the feasibility of the method we regrettably didn’t perform any assessments on tissue permeability. Possible methods to accurately quantify edema formation and alveolar fluid clearance (AFC) would have been to measure wet/dry ratio of tissue samples or to measure albumin shifting across lung compartments. All these hypotheses have to be addressed in future approaches but according to published reports and to our best knowledge, a healthy donor lung with minimal ischemic time and without structural impairments can be safely perfused for a prolonged period exceeding the duration reached in this study^12,15,45^.

Even in best possible surgical conditions ischemia/reperfusion injury can never be completely avoided in our model. Nonetheless a learning curve was observed and further improvements in our technique will lead to shorter and less variant ischemic times in future experiments, thus improving comparability between each case.

Despite the above-mentioned challenges, another limitation is given by the relatively small sample number. Having been designed as a pilot trial to future comparative experiments, this series does not allow a statistical correlation between ILP related variables and tumor related clinicopathological parameters. Reasonably, size and the histopathological characteristics might correlate with parameters of metabolism during ILP. Measurements of biomarkers in the tissue, perfusate and exhalate may provide further insights in tumor biology and provide a basis for *ex vivo* testing of treatment approaches in future settings.

Importantly, as previously postulated^4^, no disadvantages arose for the patient in performing an ILP on the resected lobe. Intraoperative evaluation of the resection margins as well as histological diagnosis and tumor staging remained unaffected by the procedure. Frozen sections were taken before ILP and the tissue specimen remained vital throughout the experiment.

Taken together, this series successfully demonstrated that ILP of lung lobes is a feasible approach in which all functional parameters could be precisely assessed and kept steady over time, thereby allowing for unmitigated comparative analyses. Although previously addressed in the context of EVLP^46–48^, this series is to our best knowledge the first one to present genuine data on glucose metabolism of isolated perfused lung lobes. The calculated glucose consumption of 1.86 mg/min/LTLC (95% CI: −2.09 to −1.63) is similar to the one observed in marginal donor lungs −2.03 ± 1.03 mg/min/LTLCp, substantiating the reliability of the novel approach.

## Conclusion

In summary, the innovative technique of isolated lung perfusion on human pulmonary lobes which have been surgically resected due to underlying malignancy allows to mimic physiological conditions in a highly controlled environment for up to 6 hours. By the plethora of possible applications in lung specific experimental research this novel approach has the potential to reduce the need for animal testing and to save up on research resources. Our findings may pave the way for future personalized approaches not only for patients with primary lung cancer but also for patients with pulmonary metastases undergoing resection.
